# Comprehensive landscape of subtype-specific coding and non-coding RNA transcripts in breast cancer

**DOI:** 10.18632/oncotarget.11998

**Published:** 2016-09-13

**Authors:** Trung Nghia Vu, Setia Pramana, Stefano Calza, Chen Suo, Donghwan Lee, Yudi Pawitan

**Affiliations:** ^1^ Department of Medical Epidemiology and Biostatistics, Karolinska Institutet, SE 17177 Stockholm, Sweden; ^2^ Department of Molecular and Translational Medicine, University of Brescia, 25125 Brescia, Italy; ^3^ Department of Statistics, Ewha Womans University, Seodaemun-gu, Seoul 120-750, South Korea

**Keywords:** breast cancer, RNA sequencing, subtype-specific isoforms, subtype co-expression, non-coding RNAs

## Abstract

Molecular classification of breast cancer into clinically relevant subtypes helps improve prognosis and adjuvant-treatment decisions. The aim of this study is to provide a better characterization of the molecular subtypes by providing a comprehensive landscape of subtype-specific isoforms including coding, long non-coding RNA and microRNA transcripts. Isoform-level expression of all coding and non-coding RNAs is estimated from RNA-sequence data of 1168 breast samples obtained from The Cancer Genome Atlas (TCGA) project. We then search the whole transcriptome systematically for subtype-specific isoforms using a novel algorithm based on a robust quasi-Poisson model. We discover 5451 isoforms specific to single subtypes. A total of 27% of the subtype-specific isoforms have better accuracy in classifying the intrinsic subtypes than that of their corresponding genes. We find three subtype-specific miRNA and 707 subtype-specific long non-coding RNAs. The isoforms from long non-coding RNAs also show high performance for separation between Luminal A and Luminal B subtypes with an AUC of 0.97 in the discovery set and 0.90 in the validation set. In addition, we discover 1500 isoforms preferentially co-expressed in two subtypes, including 369 isoforms co-expressed in both Normal-like and Basal subtypes, which are commonly considered to have distinct ER-receptor status. Finally, analyses at protein level reveal four subtype-specific proteins and two subtype co-expression proteins that successfully validate results from the isoform level.

## INTRODUCTION

One in eight women will develop an invasive breast cancer during their lifetime and, despite the implementation of screening and prevention programs [1], more than 131,000 women died of breast cancer in Europe in 2012 [2] and approximately 40,000 deaths are expected in the United States in 2016 [3]. These, respectively, represent the first and second most-common cause of cancer-related deaths among women. Reasons include the fact that breast cancer is such a complex and heterogeneous disease in terms of molecular alterations and clinical outcomes [4] that it should be considered not as a single disease but rather as a group of molecularly distinct neoplasms [5].

In the last decade many studies have investigated the distinct breast-cancer subtypes through their characteristic molecular profiles, and their clinical correlation to prognosis and response to therapy. These molecularly defined subtypes differ in expression of well-known and therapeutically important receptors: estrogen receptor (ER) and human epidermal growth factor receptor 2 (HER2). Based on gene expression signatures at least five independent intrinsic molecular subtypes: Normal-like, Luminal A and B (mostly ER+), Basal (mostly ER- and HER2-) and Her2/ERBB2 (mostly ER- and HER2+) have been consistently reported in different cohorts [6–10]. When progesterone receptor (PR) expression is also considered, the Basal subtype is close, though not identical, to the so-called triple-negative subtype. Luminal A cancers, exhibiting high ER and PR expression, are early stages and have generally better prognosis, with little extra benefit from conventional chemotherapy. On the other hand, Basal cancers have poor prognosis and tend to show low levels of all three receptors, leading to limited options for targeted therapy, but they seem to respond to conventional chemotherapy [11]. While molecular subtyping has been translated in an individualized therapeutic approach, we are still far from the implementation of an effective personalized medicine [12].

Recent development of next generation sequencing (NGS), especially in transcriptomics through RNA-sequencing, provides an accurate estimation of transcript- or isoform-level expression and potentially gives more insight into the disease. More than 95% of human genes encode splice variants [13], and even though isoforms of the same gene may produce proteins which are very similar in their sequence, differences induced by alternative splicing can influence the function of the variants [14, 15]. Indeed some variants might exert non-redundant and sometimes antagonistic functions and might have a substantially different association with tumor characteristics [16–20]. For example, comparing the different isoforms of p53 (*TP53*) in breast cancer, Avery-Kiejda *et al.* [20] observed that isoform *D40p53* was up-regulated in tumor compared to normal tissue, and was associated with an aggressive triple negative subtype. On the other hand, over-expression of isoform *p53b* was associated with less aggressive tumors with smaller size and longer disease-free survival. In a recent paper [21], *PELP1*, a well-known proto-oncogene whose dysregulation is implicated in oncogenesis and therapy resistance, was found to be involved in alternative splicing modulation, which in turn might lead to the activation of pathways supporting tumor progression.

Despite the growing evidence that isoform-level expression pattern might be more informative than gene-level expression, at present little work has been done towards characterization of breast-cancer subtypes using genome-wide isoform-level expression data [22]. The Cancer Genome Atlas (TCGA) has profiled a large number of breast cancers at DNA, RNA and protein levels using several platforms. As reported in Koboldt et al [23], the integration of multiple sources of information confirmed the existence and assisted the further characterization of the intrinsic subtypes. However, while providing new insights into the subtype molecular profiles, the work by Koboldt et al still relied on mRNA quantification at gene level. To our knowledge, all studies that explored breast-cancer subtypes based on genome-wide mRNA expression have done so at gene level. Although several researches [24–27] investigated the isoform-level expression in breast-cancer subtypes, their studies usually focus on isoforms of single genes, rather than whole transcriptome-wide analyses.

Non-coding RNAs [28] such as microRNAs (miRNAs) and long non-coding RNAs (lncRNAs) do not encode proteins, but they are highly involved in gene regulation. MiRNAs are well-known, but the role of lncRNAs in different human cancers [29, 30], including breast cancer [31], have also been widely investigated. In contrast to miRNAs, lncRNAs are commonly defined as non-protein coding molecules longer than 200 nucleotides [32]. Thus the main aim of this article is to take advantage of RNA-seq data of 914 TCGA breast cancer samples to provide a comprehensive molecular subtype-specific characterization in terms of isoform-level expression of all mRNA and lncRNA, and miRNA transcripts. We particularly aim to establish the subtype-specificity and the subtypes co-expression patterns.

## RESULTS

**Figure 1:**
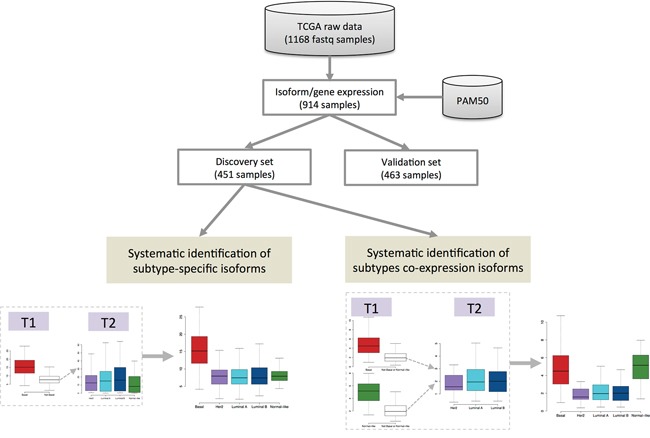
Pipeline of systematic identification of subtype-specific isoforms and subtype co-expression isoforms from breast cancer TCGA RNA-seq data T1 is a statistic to compare a single subtype against all other subtypes. Statistic T2 is used to compare the corresponding other subtypes to each other.

An isoform is said to be specific to a single subtype if it satisfies these two conditions: (i) it is significantly over-expressed in that subtype compared to all the other subtypes, and (ii) the other subtypes cannot be separated based on that isoform. If we have more than two subtypes, differential expression alone is not enough to guarantee subtype-specificity. “Subtypes co-expression” is an extension of subtype-specificity, where we consider a pair of subtypes with similar expression for a particular isoform. The pipeline for systematic identification of subtype-specific isoforms and subtype co-expression patterns is presented in Figure 1. More details are given in Materials and Methods.

### Subtype-specific isoforms analysis

#### Examples of isoform-level distributions

**Figure 2:**
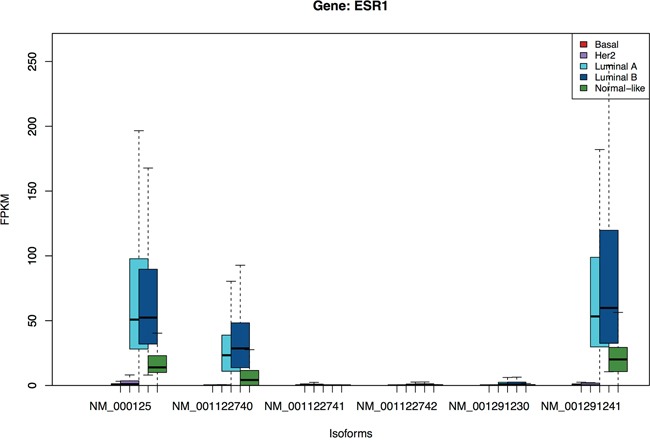
Isoform-level expression distribution of *ESR1* gene across 5 molecular subtypes X-axis labels are transcripts ids. The figure shows that *ESR1* expression is mostly contributed by three isoforms: *NM_000125*, *NM_001122740* and *NM_001291241*, and these isoforms have similar expression patterns.

To highlight the value of isoform-level information, Figure 2 shows the boxplots of the isoform expression of the estrogen receptor-alpha *ESR1*, a fundamental gene in breast cancer biology. The figure shows that *ESR1* expression is dominated by three isoforms: *NM_000125*, *NM_001122740* and *NM_001291241*. These isoforms have similar expression patterns, with high expression in Luminal A and B, low expression in Normal-like and almost no expression in Basal and Her2 subtypes. The pattern of these isoforms is more obvious in the color map given in the Supplementary Figure S1, where they present a highly positive correlation.

**Figure 3:**
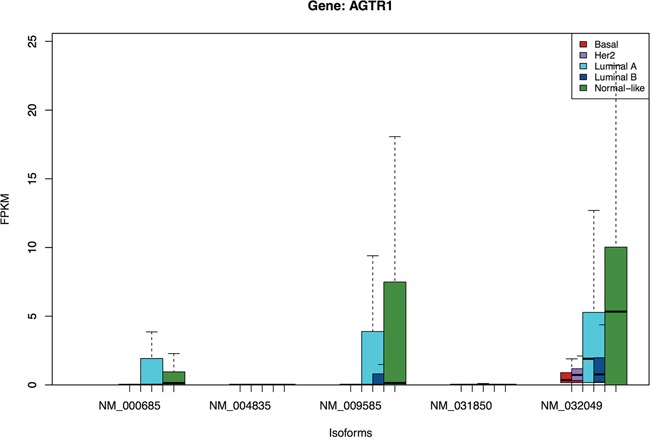
Boxplots of isoforms of gene *AGTR1* Three isoforms in this gene are over-expressed in primarily the Luminal A and Normal-like, but not in the Luminal B subtype.

From Figure 3, three isoforms of *AGTR1* (angiotensin II receptor, type 1) are over-expressed in primarily the Luminal A and Normal-like, but not in the Luminal B or other subtypes. The gene codes for a potent vasopressor hormone and a primary regulator of aldosterone secretion. Recently Rhodes et al [33] reported that *AGTR1* overexpression defines a subset of estrogen-receptor (ER)-positive breast cancer and confers sensitivity to losartan, an *AGTR1* antagonist. Our analysis here shows that the expression is specific to the luminal A and Normal-like subset of the ER-positive patients, but not the luminal B, and that the isoform *NM_032049* is likely the best marker for this subgroup.

In general there is a large heterogeneity in the expression profiles across the isoforms of the same gene, as well as across different subtypes. Therefore there might potentially be additional information hidden at isoform-level expression.

#### Identification of subtype-specific isoforms

**Figure 4:**
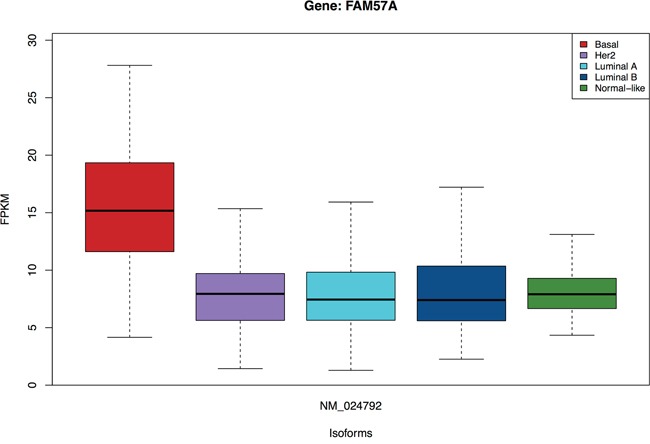
Isoform *NM_024792* of the *FAM57A* gene is specific for the Basal subtype

The subtype assignment of the TCGA samples is used for identifying subtype-specific isoforms. Here, we are interested in isoforms which are not only highly significant in one group/subtype, but also have similar expression across the rest of the groups/subtypes. An example is presented in Figure 4, where the isoform *NM_024792* from gene *FAM57A* is over-expressed in the Basal subtype but has 2-fold lower expression across the rest of the subtypes. We identify 4960 subtype-specific isoforms with t-statistics FDR< 0.01, chi-squared FDR> 0.10. Besides, we compensate for the difference between the four remaining subtypes (allowing a slightly lower chi-squared FDR) by the fold-change between medians of the specific subtype and the rest. We discover 469 additional subtype-specific isoforms with t-statistics FDR< 0.01, chi-squared FDR> 0.01 and fold-change > 5. The list of a total of 5451 subtype-specific isoforms is available in Additional file 3. These isoforms are from 4533 genes of which 67.02% are multiple-isoform genes. A summary of the number of isoforms per gene from this gene list is presented in the Supplementary Figure S2.

The top 5 isoforms of each subtype are given in Table 1. For these top 5 mRNA isoforms, the Normal-like subtype achieves a median AUC of 0.96, and this also obtains the highest median AUC of 0.92 in the validation set (Table 2). The Her2 and Basal- have median AUCs of >0.87, while the lowest median AUC of 0.76 is achieved by the Luminal A. The results of lncRNA isoforms are similar to mRNA isoforms but slightly lower AUCs for all subtypes.

**Table 1:** Top 5 subtype-specific isoforms for each subtype

Subtype	mRNA isoforms	lncRNA isoforms
Basal	*NM_173587(RCOR2),NM_178562(TSPAN33), NM_021154(PSAT1),NM_058179(PSAT1),NM_003412(ZIC1)*	*NR_026877(MGC2889),NR_120532(LOC101929427),NR_027793(LINC00518),NR_002947(TCAM1P),NR_028406(FXYD5)*
Her2	*NM_001085437(C2orf54),NM_001291730(PGAP3),NM_001030002(GRB7),NM_001165938 (STARD3),NM_001165937(STARD3)*	*NR_103466(NBPF13P),NR_110717(LINC01351),NR_109896(MFSD2A),NR_110167(LINC01213),NR_072994_6(PPP1R10)*
Luminal A	*NM_001083536(FGD3),NM_001378(DYNC1I2), NM_001193288(SLC24A2),NM_198485(TPRG1),NM_001160173(NAT1)*	*NR_036537(RAB6C-AS1),NR_109862(LOC100506674),NR_024559(MAPT-AS1),NR_104018(PMP22),NR_040090_5(CYP21A1P)*
Luminal B	*NM_000946(PRIM1),NM_020748(INTS2),NM_198463(C3orf67),NM_181725(METTL2A),NM_021215(RPRD1B)*	*NR_038896(DSCAM-AS1),NR_038900(DSCAM-AS1),NR_038899(DSCAM-AS1),NR_038341(STK4-AS1),NR_024063(ZSCAN12P1)*
Normal-like	*NM_002380(MATN2),NM_001130005(ACTN1),NM_021902(FXYD1),NM_002055(GFAP),NM_173833(SCARA5)*	*NR_024011(LOC286367),NR_108046(LINC008 44),NR_001284(TNXA),NR_024359(LINC00086),NR_015423(PGM5-AS1)*

**Table 2:** Median AUC of the top 5 subtype-specific isoforms in the discovery and validation sets

	mRNA		lncRNA	
	Discovery	Validation	Discovery	Validation
Basal	0.93	0.88	0.88	0.87
Her2	0.87	0.81	0.78	0.65
Luminal A	0.76	0.72	0.75	0.75
Luminal B	0.78	0.72	0.75	0.68
Normal-like	0.96	0.92	0.96	0.90

**Figure 5:**
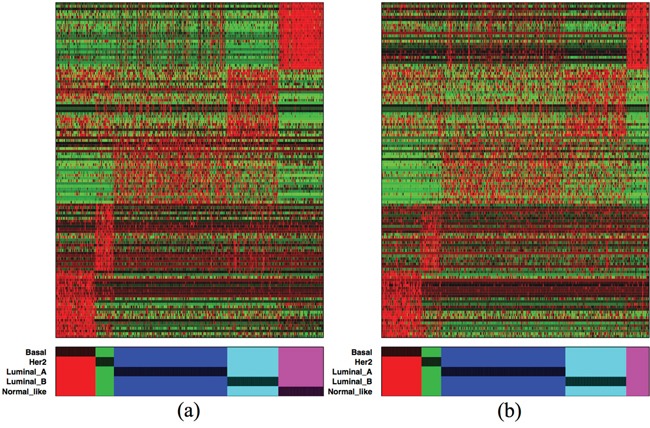
Color-map of the top 125 subtype-specific isoforms (25 from each subtype) from (a) the discovery and (b) validation sets Red and green indicate expression levels above and below median, respectively. The isoforms in each subtype are ordered by AUC from bottom to top and right to left.

The color map of the top 125 isoforms (25 isoforms from each subtype) in the discovery set is presented in Figure 5(a). A similar pattern can be observed in Figure 5(b) from the validation set. These 125 isoforms come from 113 genes, so only a few isoforms from the same genes contain similar information. Thus there is no great redundancy of information among the isoforms from the same gene, but it also means that for these marker genes it is often sufficient to consider a single dominant isoform. We observe that the Basal- and Normal-like- specific isoforms are the most distinct expression in both the discovery and validation sets, although the Her2-specific isoforms found in the discovery set are also observed in the validation set. This is not the case for the Luminal A- and Luminal B-specific isoforms, where the expression of the isoforms in the validation data seems not as high as in the discovery set. The list of these 125 subtype-specific isoforms is given in the Additional File 4.

**Figure 6:**
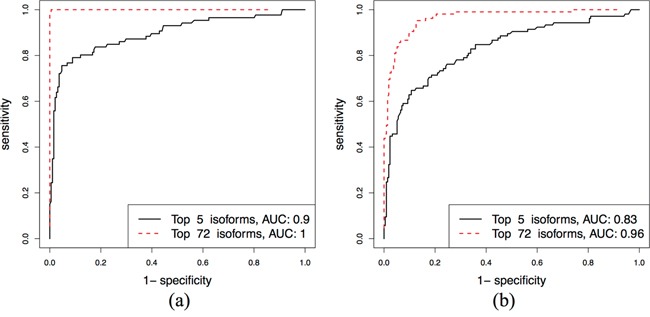
Separating Luminal A and Luminal B subtypes: ROC curves for the top 5 and 74 isoforms in the discovery and validation sets

The Luminal A and B subtypes are the most easily confused with each other, but there are many potential subtype-specific isoforms. So, to distinguish these two subtypes, we use a panel of rather than single isoforms, starting with 1807 isoforms that are specific for Luminal A and B. Using penalized L1-logistic regression method, 72 isoforms are selected. To further refine the variable selection, isoforms are sorted in the order of their regression coefficients, and the AUCs of ROC curves are calculated from fitted values of logistic regression (that can be considered as composite marker) for an increasing number of features included in the model; see Supplementary Figure S3. The ROC curves in Figure 6 show that 5 isoforms give sufficiently high performance (AUC = 0.90) to distinguish the Luminal A and B. The AUC in the validation dataset is 0.96 using a set of 72 isoforms.

Classification using the corresponding gene-level expression is also performed. The median AUC of isoform-level expression (0.65) is slightly lower than that of gene-level expression (0.67), see Supplementary Table S3. Note that to compare with isoform-level classification we must account for the level of contribution of the isoform to the total gene expression. Indeed as we expect, when a few dominant isoforms are the main contributors of the corresponding gene expression, the isoform- and gene-level classification will perform similarly; see Supplementary Figure S4. As illustrated by the *ESR1* gene (Figure 1), this does not mean that isoform-level data carries no additional information, since even in this case we may still gain more specific biological information. Also from Supplementary Figure S4, for less dominant isoforms, we observe greater differences in classification performance. Of the subtype-specific isoforms, 27% (1468) have better classification than their genes. Focusing on the top 10% isoforms with large differences in isoform- and gene-level AUCs, we then check if they perform similarly in the validation set. For instance, for the Basal subtype, from 103 isoforms with AUC difference larger than 90th percentile, 68 isoforms show similar pattern in the validation set (Supplementary Table S4).

#### Subtype-specific miRNA and lncRNA isoforms

We first investigate subtype-specific isoforms from the miRNA molecules, which are short non-coding RNA of about 21-25 nucleotides in length. We find three isoforms from miRNAs identified as subtype-specific (Supplementary Figure S5). Two isoforms *NR_024607* and *NR_001458* are the unique isoform of genes *MIR503HG* and *MIR115HG* respectively. Gene *MIR22HG* contains isoform *NR_028503* and three others. Most of the isoforms have low expression, with over-expression in Normal-like and Basal subtypes only. However, these patterns are also consistent with previous studies at gene-level. For example, in a study of 51 human breast cancer cell lines [34], *MIR22HG* and *MIR155HG* are demonstrated to be significantly higher expressed in normal-like cells and basal-like cells respectively. Another recent study [35] suggests that expression of *MIR503HG* is significantly downregulated in breast cancer tissues and cells.

A total of 707 lncRNA isoforms are subtype-specific. We keep 210 isoforms that are specific for Luminal A and B and select 40 isoforms by using penalized L1-logistic regression. Supplementary Figure S6 shows that the top 5 lncRNA isoforms achieve worse AUC than the top 5 isoforms of full transcriptome in the previous section, but still high AUC=0.84 in the discovery set and AUC=0.77 in the validation set. The full set of 40 isoforms obtains a greater performance with a high AUC (0.90) in validation set. Thus, the lncRNAs are also potentially markers of separation between the Luminal A and B subtypes. We take isoforms of gene *DSCAM-AS1* (Supplementary Figure S7) in the top 5 lncRNA isoforms in Luminal B subtype for further discussion. That three of the four isoforms of gene *DSCAM-AS1* are in the top 5 lncRNA isoforms indicates a strong signal in the gene. In a recent publication [36], Miano et al. studies these isoforms to investigate the relation of gene *DSCAM-AS1* with Estrogen Receptor alpha (ERα) in luminal breast cancer cells. The results show that the four isoforms are similarly down-regulated after the silencing of Estrogen Receptor alpha (ER α). Moreover, the gene expression is significantly higher in Luminal B and they report *DSCAM-AS1* as a major discriminant of the luminal subtype in breast cancer [36].

### Subtype co-expression patterns

Co-expression of the same isoform by two subtypes may reveal some underlying molecular similarities; for example, the Basal and Her2 subtypes have low expression of estrogen receptor gene. We discover 1500 isoforms that are co-expressed in two subtypes (t-statistic FDR< 0.01 and chi-squared test FDR> 0.1); the full list is in Additional file 5. It is interesting that most of isoforms, 9 of 12, in the top 12 (ranked by t-statistics) in Supplementary Figure S8 are highly expressed in the Basal and Normal-like subtypes, despite the fact that these subtypes have typically distinct ER-receptor status. Of the remaining three, isoform *NM_013409* from gene *FST* overexpresses in Luminal A and Normal-like subtypes, and two other isoforms *NM_004923* of *MTL5* and *NM_017843* of gene *BCAS4* have overexpression in Luminal A and Luminal B subtypes.

We further investigate isoforms belonging to genes *LDHB* and *FST* from the top 12 isoforms, as they have been previously reported at gene level. The boxplots of isoform-level expression of these genes are presented in Supplementary Figures S9 and S10, respectively. Gene *LDHB* encodes for the lactate dehydrogenase B protein that was highly expressed in Basal subtype and reported to be a metabolic marker of response to neoadjuvant chemotherapy in breast cancer [37]. This gene has 2 isoforms that are dominated by isoform *NM_002300*; both are under-expressed in the Her2, Luminal A and Luminal B groups. For the follistatin gene *FST*, Bloise et al. [38] suggest a role in benign breast disease through a differential expression in stromal cells. The role of *FST* in breast disease is mentioned again in a recent study [38] on breast cancer patients. Two isoforms *NM_006350* and *NM_013409* show high expression in Luminal A and Normal-like subtypes, and the former is likely the best marker for this co-expression pattern.

Supplementary Table S5 shows that most of the significant isoforms are Luminal A–Normal-like specific. We sort the isoforms based on the t-statistics, and for groups with more than 25 isoforms the best 25 are selected. A color map of these genes in the discovery and validation sets is presented in Supplementary Figure S11. Distinct profiles of the Basal–Normal-like, Luminal A–Luminal B, Luminal A–Normal-like and Basal-Luminal B specific isoforms can be clearly observed. The same patterns can be seen also in the validation set.

### Subtype-specific validation by protein expression

The same analysis is then applied to systematically identify subtype-specific proteins from a TCGA protein-expression dataset. The dataset generated using Reverse Phase Protein Array (RPPA) includes 280 proteins from 668 patients. The distribution of subtypes in the protein validation set is presented in Supplementary Table S2. We discover 38 subtype-specific proteins. Four of these proteins validate the results from subtype specific isoforms by sharing the same corresponding genes and specific subtypes. Specifically, the proteins encoded by genes *G6PD*, *ACACA*, *CDKN2A* and *ARAF* are specific to Her2, Luminal B, Basal and Luminal A, respectively.

**Figure 7:**
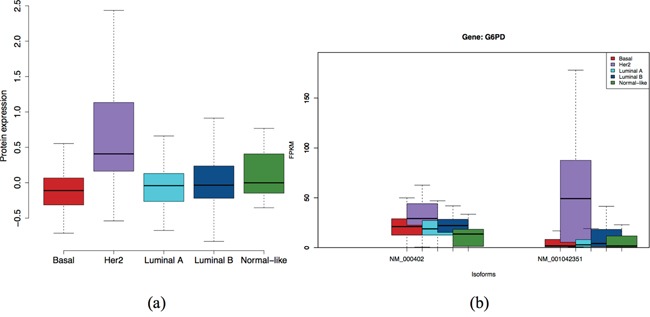
Expression at protein level **a.** and isoform level **b.** of *G6PD* gene. Isoform *NM_001042351* and the protein are specific to Her2 subtype.

Gene *G6PD* encodes glucose-6-phosphate dehydrogenase, which is an important cytosolic enzyme involved in a metabolic pathway leading to cellular reducing energy in the form of NADPH [39]. In Figure 7, Her2 subtypes of these proteins and isoforms of gene *G6PD* are significantly overexpressed to the other subtypes. The plots of other genes are also available in Supplementary Figures S12-S14 of the Supplementary report. As reported in GeneCards [40], there is high confidence that G6PD protein is found in extra-cellular compartment (confidence=5), and fairly high confidence (confidence=4) it is in the cytosol and membrane. Furthermore, it is normally not found in serum or plasma. So if the over-expressed G6PD protein in HER2 tumors is leaked out to the blood stream, it is an excellent candidate for a serum or plasma-based marker.

Finally, we explore 14 subtype co-expression proteins and find that two have the same pair of overexpressed subtypes to at least one isoform of the corresponding genes: *ESR1* for Luminal A and Luminal B subtypes (Supplementary Figure S15) and *MYH11* for Luminal A and Normal-like subtypes (Supplementary Figure S16).

### Availability

The results of subtype-specific isoform analysis in this research are available for use in an interactive website at https://nghiavtr.shinyapps.io/BRCAsubtypes/.

## DISCUSSION

We have demonstrated a detailed analysis of isoform-level patterns based on 914 RNA-seq TCGA samples in relation to the known intrinsic molecular breast cancer subtypes. Using a novel statistical methodology, we have systematically identified and validated numerous subtype-specific isoforms. Many of the subtype-specific isoforms are shown to give better accuracy in classifying intrinsic subtypes than that obtained using the whole-gene expression. We highlight the fact that expression profiles across the isoforms of the same gene vary substantially. So, particularly for key breast-cancer genes, such as *ESR1*, isoform-level data are more informative than gene-level data, allowing us to determine how variants contribute to the hormone dependence and treatment response of the tumor [41, 42].

For non-coding RNAs, we discover three miRNA isoforms that are overexpressed in the Basal and Normal-like subtypes. In addition, we find 707 long non-coding RNAs that are subtype-specific isoforms. The long non-coding RNA isoforms perform well in classification between two subtypes Luminal A and Luminal B.

Regarding subtypes co-expression, for the most part the same isoforms express similarly in subtypes with similar characteristics, such as the Luminal A and Normal-like. However, isoforms that are over-expressed in two rather distinct subtypes, such as the Normal-like and Basal, are worth investigating further. The hormone-sensitive tumors Luminal A and B have different treatments, but the histopathological diagnosis of these tumors is difficult. We have identified a panel of classifier isoforms that can classify these two types with high power in both discovery and validation sets. Intriguingly we have found very limited numbers of isoforms that are co-expressed in the Basal–Luminal A or Her2–Luminal A pairs, indicating deep molecular distinction between these pairs of subtypes.

Validation at protein level reveals four proteins that have at least one isoform of the corresponding genes sharing the same specific subtypes. Moreover, G6PD protein which is specific to HER2 subtype, is potentially an excellent candidate for serum or plasma-based marker. For subtype co-expression, two proteins also validate the results from the isoform-level analysis.

Our RNA-seq study has strengths and weaknesses. As far as we know this is the largest study of comprehensive isoform-level analysis of breast cancers. As its weakness, we have to rely on the existing transcriptome annotation, which is incomplete. This problem can be solved in the future when the technology, particularly the read lengths, improves, so that instead of relying on transcriptome annotation, one can perform transcriptome assembly of each genome. In addition, the study also might suffer the common issue of the accuracy of current quantification methods for hundreds of genes described in a recent study [43]. The accuracy mainly depends on the degree of the unique information that allows mapping of the reads to the correct transcripts and genes. In that report [43], the authors discovered 958 problematic transcripts/genes that are difficult to measure accurately. Fortunately, none of them are in the top discovered subtype-specific or subtype-coexpression isoforms in our study. Although Sequgio used in our study shows better accuracy than many common isoform quantification methods [44], issue of the problematic genes might still happen. For convenience, we supply the information of problematic isoforms in the RShiny app so that users can make further checks.

We have more detailed characterization of the intrinsic molecular subtypes based on isoform-level expression of all coding and non-coding RNAs. Since identification of the molecular subtypes helps prognosis and therapy decision, this isoform-level information may be used as therapeutic markers.

## MATERIALS AND METHODS

### TCGA RNA-sequence data

The raw data in this study comprise 1168 invasive breast carcinoma (BRCA) downloaded (June 2014) from The Cancer Genome Atlas (TCGA) after approval from the TCGA data access committee. All TCGA samples have been collected following strict human subjects protection rules, informed consent and Institutional Review Board approval of the protocols; see the project website at http://cancergenome.nih.gov for more details. We keep 1137 unaligned samples after eliminating all tissues that are sequenced more than once. The samples come from 29 different hospitals, but were all sequenced at the University of North Carolina (UNC) using Illumina HiSeq. This platform uses paired-end reads with read length of 50 bp, and 6-100 million reads were generated per sample. Reads were mapped to human hg19 UCSC annotation reference using Tophat [45] and Bowtie [46] to create the bam files.

Isoform-level expression is estimated using Sequgio analysis pipeline [44] (following the instruction of its webpage http://fafner.meb.ki.se/biostatwiki/sequgio/), which reports expression values in units of fragments per kilobase of transcript per million mapped reads (FPKM). Gene-level FPKM is defined as the sum of the isoform-level expression. We extract the list of all non-coding RNAs from the reference annotation and define the transcripts longer than 200 nucleotides as lncRNA isoforms. There is a total of 48,009 RNA isoforms in the reference annotation, including 37,990 coding mRNAs, 7649 lncRNAs, 1755 miRNAs and 615 other non-coding RNAs. After removal of 5 potential outliers using the principal component analysis (PCA) plot and 218 samples with missing PAM50-subtype information, the final dataset contains 914 samples. PAM50 is a minimal gene set derived by Parker et al. [4] for classifying “intrinsic” subtypes of breast cancer and commonly used for breast cancer subtype research including the TCGA breast cancer project. In the TCGA breast cancer project, the 50-gene PAM50 model [4] is applied to mRNA expression data to classify each sample [47]. These samples are then split, according to sample-tissue source, almost equally into a discovery set (n=451) and a validation set (n=463) as shown in Supplementary Table S1. The distributions of the molecular subtypes in both discovery set and validation set (Supplementary Table S2) are similar. The discovery set is used for systematic identification of subtype-specific isoforms and subtype co-expression patterns; see Figure 1 for an overview.

### Systematic identification of subtype-specific isoforms

At gene-level expression, a gene may be over-expressed in a specific subtype. However, this gene-level information may not translate to all isoforms of the gene. Expression of the gene could be dominated by a few isoforms, or generally different isoforms within the gene may behave differently. So isoform-level information from the RNA-seq data allows us to investigate deeper into the biology of the molecular subtypes. Identifying subtype-specific isoforms, which are significantly over-expressed in a single subtype compared to the rest of the subtypes, is the next challenge. To be ‘subtype-specific’ it is not enough for the isoform to be over-expressed in one subtype, but it must also be the case that we cannot distinguish the other subtypes based on that isoform. The ideal subtype-specific isoform is one that is expressed in one isoform only (i.e. no expression otherwise), but for a comprehensive analysis we need a less strict definition.

The overview of the analytic pipeline is presented in Figure 1. When there are only two subtypes, systematic identification of subtype-specific isoforms is a standard statistical problem, i.e. by differential expression analysis. However, the problem is more difficult if we have more than two subtypes. If we simply test one subtype, say A, against all the others, a significant result does not imply that the isoform information is specific to subtype A. It only means that subtype A is different from all the others, but the rest could also be different from each other. So, to establish this subtype specificity, we also need to test that the other subtypes are not statistically different from each other. Thus two statistical tests are required. Specifically, for each isoform, we first fit a one-way ANOVA model assuming quasi-Poisson outcomes [48]. We then compute two statistics for each subtype:

- T1 that compares each single subtype against all other subtypes,
- T2 that compares the corresponding other subtypes to each other.

For example, suppose there are 5 subtypes A, B, C, D and E; then we compute 5 pairs of statistics (T1,T2). The first T1 compares A vs (B,C,D,E), while the corresponding T2 jointly compares B, C, D and E. To get a subtype-specific isoform, T1 must be large but at the same time T2 must be small. More detailed description of the statistical method is presented in the Supplementary Methods.

As expected, there are many outlying FPKM values as well as zero values, so the use of a robust regression method is crucial. We find that standard robust regression procedures (such as rreg in R) fail to converge in a large proportion (roughly half) of the isoforms. Hence, we develop a new robust regression procedure for quasi-Poisson outcomes and implement it using an iterative weighted least-square (IWLS) algorithm [48]. The algorithm is highly stable and works for all isoforms. Once the robust estimates and their standard errors (or estimated variance matrices) are obtained, we compute the two-statistics as explained above: in this case T1 is the robust t-test and T2 is the chi-squared test. To account for multiple testing, the significance is expressed in terms of false discovery rate (FDR). See the Supplementary Methods for more details.

### Systematic identification of subtypes co-expression

By ‘subtypes co-expression’ we search for a pair of subtypes with similar expression for a particular isoform. For example, it is well known that the Basal and Her2 subtypes are characterized by low ER gene expression. So the subtype co-expression profile could reveal interesting biological information. The methodology is an extension of the method for the single subtype-specific isoform above, also given in the Supplementary Methods. The R codes of the statistical tests for both subtype-specific isoforms and subtype co-expression isoforms are supplied in the Additional file 6.

### Isoform-based subtype classification

The subtype-specific isoforms obtained in the previous section are then used for classifying the corresponding subtype using multiclass classification based on one-vs-all logistic regression, where every subtype is in turn compared with the remaining ones. The performance of each isoform is evaluated using the area under the receiver operating characteristic curve (AUC). For comparisons, the same procedure is also computed using gene-level expression. Although the Luminal A and B tumors have similar characteristics, particularly since both are ER+ tumors, they have different prognosis and are treated differently, so it is crucial to separate the two. Instead of using a single isoform for classification, a panel of isoforms is selected. We use L1 penalized logistic regression to select isoforms which can distinguish the Luminal A and B subtypes.

## SUPPLEMENTARY MATERIALS DATA



## Abbreviations

ANOVA: analysis of variance
AUC: area under the curve
BRCA: breast carcinoma
ER: estrogen receptor
FPKM: fragments per kilobase of transcript per million mapped reads
HER2: human epidermal growth factor receptor 2
IWLS: iterative weighted least-square
NGS: next generation sequencing
PR: progesterone receptor
RNA-seq: RNA Sequencing
ROC: receiver operating characteristic
TCGA: The Cancer Genome Atlas
FDR: False Discovery Rate
TP53: tumor protein p53
ESR1: estrogen receptor 1
LDHB: lactate dehydrogenase B
FST: follistatin
FAM57A: family with sequence similarity 57 member A
INTS2: integrator complex subunit 2
AGTR1: angiotensin II receptor type 1
RPPA: Reverse Phase Protein Array
G6PD: glucose-6-phosphate dehydrogenase
NADPH: nicotinamide adenine dinucleotide phosphate
ACACA: Acetyl-CoA Carboxylase Alpha
CDKN2A: Cyclin-Dependent Kinase Inhibitor 2A
ARAF: A-Raf Proto-Oncogene, Serine/Threonine Kinase
MYH11: Myosin Heavy Chain 11, Smooth Muscle
DSCAM-AS1: Down Syndrome Cell Adhesion Molecule Antisense RNA 1
