# Supplementary material for: Comprehensive landscape of subtype-specific coding and non-coding RNA transcripts in breast cancer
Source: Oncotarget. 2016 Sep 13;7(42):68851–63. doi: 10.18632/oncotarget.11998 (PMC5356595; doi:10.18632/oncotarget.11998)
Supplement: Supplementary file 1 [file oncotarget-07-68851-s001.pdf]

Comprehensive landscape of subtype-specific coding and non-coding RNA transcripts in breast cancer

SUPPLEMENTARY DATA

Identifying subtype-specific isoforms

In the next generation sequencing platform, g ene expression is presented in Fragments Per Kilobase of transcript per Million mapped reads (FPKM) unit. For modeling FPKM, we use quasi-Poisson model as follows (Pawitan, 2001). y_m is FPKM of isoform m and assumed to follow a quasi-Poisson distribution $y_m \sim Poisson(\mu_m)$ with

$$\begin{aligned} E(y_m) &= \mu_m \\ Var(y_m) &= \varphi\mu_m \end{aligned} \tag{1}$$

m=1,2,...M, M is number of isoforms.

Then for each isoform we define:

$$\mu_{ij} = \mu + \beta_i + e_{ij}, \tag{2}$$

where $\sum \beta_i = 0$, μ_{ij} is median of i-th subtype, μ is general median, i=1,...,k, and k is number of subtypes. The analysis obtaining subtype-specific isoforms is performed in two steps defined as follows.

Finding the best group

In this step, we would test for

$$H_0 : \beta_1 = ... = \beta_k = 0 \tag{3}$$

In order to obtain a robust estimation, we perform iterative weighted least-squares (IWLS) (Pawitan, 2001) as the following:

1. Obtain an initial estimate from the observed data $Y_i = \mu + x_i'\beta_i + e_i^*$ where e_i^* is the working residual $e_i^* = e_i$ and the variance of e_i^* is defined: $\mathrm{var}(e_i^*) = \varphi\mu_i$

$$\mathrm{var}(e_i^*) = \begin{cases} \mathrm{var}(e_i^*) & \text{if } \mathrm{var}(e_i^*) > 0.1 \\ 0.1 & \text{otherwise} \end{cases}$$

2. Use robust weight such that residuals greater than Q_3 received smaller weights:

$$w(e) = \begin{cases} 1 & |e| \le Q_3 \\ Q_3/(|e|+0.001) & |e| > Q_3 \end{cases}$$

Therefore we can obtain the regression estimate:

$$\hat{\beta} = (X'\Sigma^{-1}X)(X'\Sigma^{-1}Y) \tag{4}$$

where **X** is a design matrix so that $\sum \beta_i = 0$ and Σ^{-1} is a diagonal matrix with element:

$$\Sigma^{-1} = \frac{1}{\mathrm{var}(e_i^*)} \times w(e_i)$$

For 5 groups, **X** is defined as

$$X = \begin{pmatrix} 1 & 0 & 0 & 0 \\ 0 & 1 & 0 & 0 \\ 0 & 0 & 1 & 0 \\ 0 & 0 & 0 & 1 \\ -1 & -1 & -1 & -1 \end{pmatrix}$$

3. Iterate step 1 and 2 until converge

After the $\hat{\beta}$ is obtained, by assuming regularity condition we expect that it is approximately normal with estimated variance:

$$Var(\hat{\beta}) = (X'\Sigma^{-1}X)^{-1}X'\Sigma^{-1}\Sigma_y\Sigma^{-1}(X'\Sigma^{-1}X)^{-1} \tag{5}$$

where the estimate for $X'\Sigma^{-1}\Sigma_y\Sigma^{-1}$ is $\sum_i \frac{(e_i^*)^2}{\mathrm{var}(e^*)} x_i x_i'$.

Thus, for each isoform we can obtain the following robust t-statistic:

$$t_m = \frac{\hat{\beta}}{se(\hat{\beta})}, \tag{6}$$

where $se(\hat{\beta})$ is standard error.

Test non-max groups

After the significantly over-expressed group is identified, the next step is to test if the mean of gene expression of the rest of the groups are similar by testing the following hypothesis:

$$H_0 : \beta_1 = ... = \beta_{k-1} \tag{7}$$

using the following chi-squared test statistic:

$$\chi_3^2 = \hat{\gamma}'(\mathrm{var}(\hat{\gamma}))^{-1}\hat{\gamma} \tag{8}$$

where

$$\mathrm{var}(\hat{\gamma}) = C.\mathrm{var}(\hat{\beta}).C',$$

and

$$\gamma = C.\beta$$

C is a design matrix according to (7) and β is a vector of the estimates defined in (4) of the non-maximum groups.

Suppose there are 5 group, and the maximum group is the last (5th) group, then γ could be defined as follows:

$$\gamma = C.\beta = \begin{pmatrix} 1 & -1 & 0 & 0 \\ 0 & 1 & -1 & 0 \\ 0 & 0 & 1 & -1 \end{pmatrix} \begin{pmatrix} \beta_1 \\ \beta_2 \\ \beta_3 \\ \beta_4 \end{pmatrix} = \begin{pmatrix} \beta_1 - \beta_2 \\ \beta_2 - \beta_3 \\ \beta_3 - \beta_4 \end{pmatrix}$$

If the maximum is not last group, for example group 2, γ is defined as:

$$\gamma = C.\beta = \begin{pmatrix} 0 & 1 & -1 & 0 \\ 0 & 0 & 1 & -1 \\ 1 & 1 & 1 & 2 \end{pmatrix} \begin{pmatrix} \beta_2 \\ \beta_1 \\ \beta_3 \\ \beta_4 \end{pmatrix} = \begin{pmatrix} \beta_1 - \beta_3 \\ \beta_3 - \beta_4 \\ \beta_4 - \beta_5 \end{pmatrix}$$

Note that since we defined before that $\sum \beta_i = 0$, hence $\beta_5 = -\beta_1 - \beta_2 - \beta_3 - \beta_4$.

Subtype co-expression isoforms

We are also interested in the isoforms that show over-expression not only in a single subtype, but also in another subtype. For this purpose, the previous approach can be implemented with a slight modification on the chi-squared test defined in (8). For each isoform, first two most over-expressed groups are selected based on the statistics defined in (6). For the rest of the the groups (e.g., 3 out of 5 groups), a similar test as defined in (8) is performed with slightly different γ.

The design matrix **C** depends on which groups are the most maximum. Suppose there are 5 groups, and the two most over-expressed groups are the 4th and 5th group, then γ could be defined as:

$$\gamma = C.\beta = \begin{pmatrix} 1 & -1 & 0 & 0 \\ 0 & 1 & -1 & 0 \end{pmatrix} \begin{pmatrix} \beta_1 \\ \beta_2 \\ \beta_3 \\ \beta_4 \end{pmatrix} = \begin{pmatrix} \beta_1 - \beta_2 \\ \beta_2 - \beta_3 \end{pmatrix}$$

If the maximum groups are either group 1,2 or 3, the γ is as follows

$$\gamma = C.\beta = \begin{pmatrix} 0 & 0 & 1 & -1 \\ 1 & 1 & 1 & 2 \end{pmatrix} \begin{pmatrix} \beta_1 \\ \beta_2 \\ \beta_3 \\ \beta_4 \end{pmatrix} = \begin{pmatrix} \beta_3 - \beta_4 \\ \beta_4 - \beta_5 \end{pmatrix}$$

SUPPLEMENTARY FIGURES AND TABLES

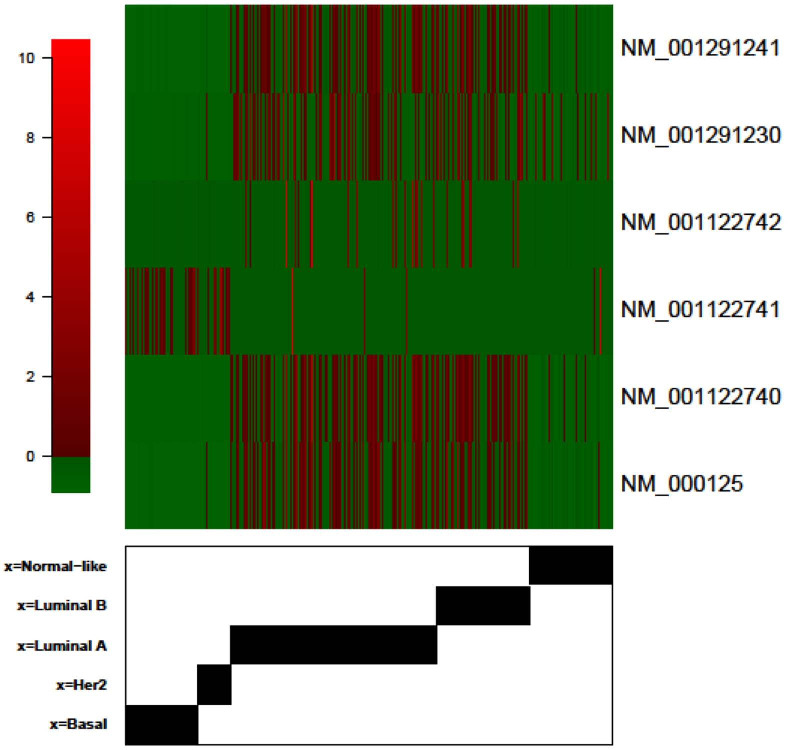

Supplementary Figure S1: Heatmap of isoforms of gene ESR1. The figure replicates the common pattern of *NM_000125*, *NM_001122740* and *NM_001291241* with high expression in Luminal A and B, low expression in Normal-like and almost no expression in Basal and Her2 subtypes.

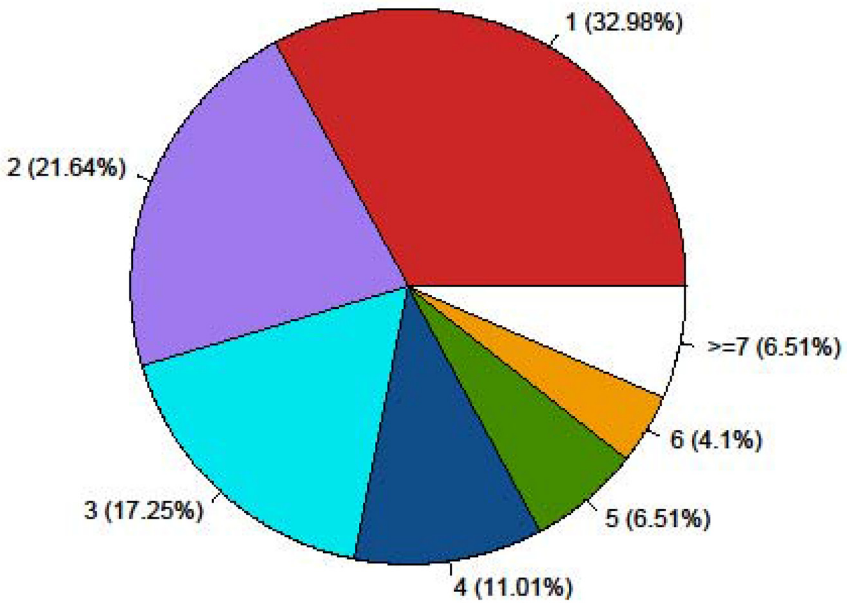

Supplementary Figure S2: The proportion of the numbers of isoforms per genes in the gene list extracted from subtype-specific isoforms.

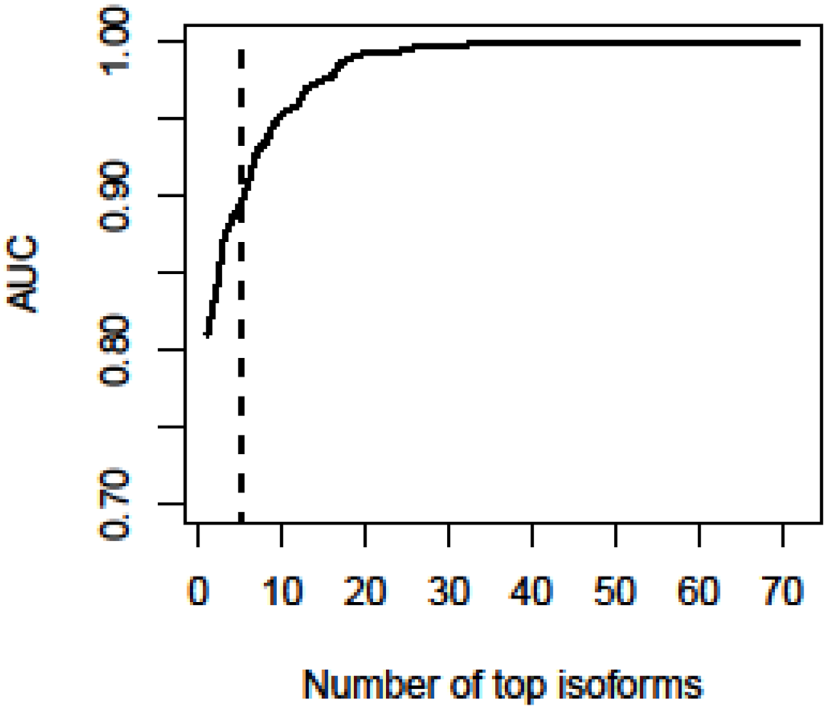

Supplementary Figure S3: AUC of the classification of Luminal A vs Luminal B subtype as a function of the number of top isoforms used in logistic regression. The dash line indicates the top 5 isoforms. In Figure 6 of the main text we plot the ROCs based on 5 and 72 isoforms.

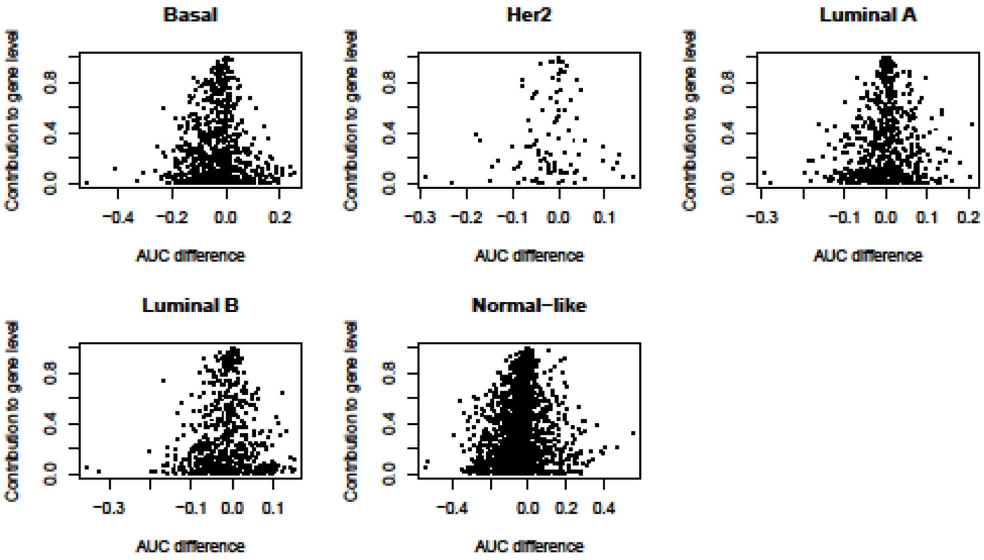

Supplementary Figure S4: Scatter plot of AUC differences (isoform-level value minus gene-level value) versus the percent contribution of the isoform to the total gene-level expression. When the contribution is low, there could be a large difference in the AUC.

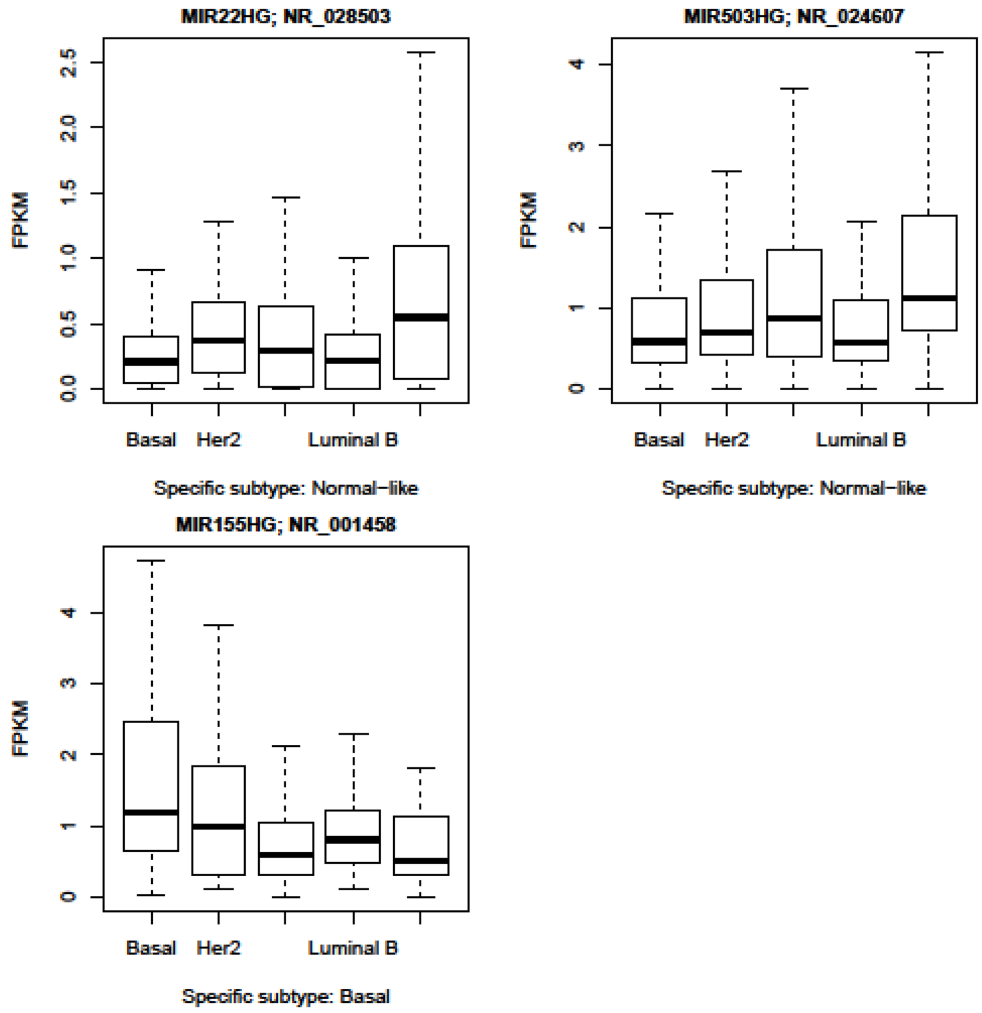

Supplementary Figure S5: Boxplots of 3 subtype-specific miRNA isoforms. On the x-axis, the subtypes follow the alphabetical order of Basal-like, Her2, Luminal A, Luminal B and Normal-like.

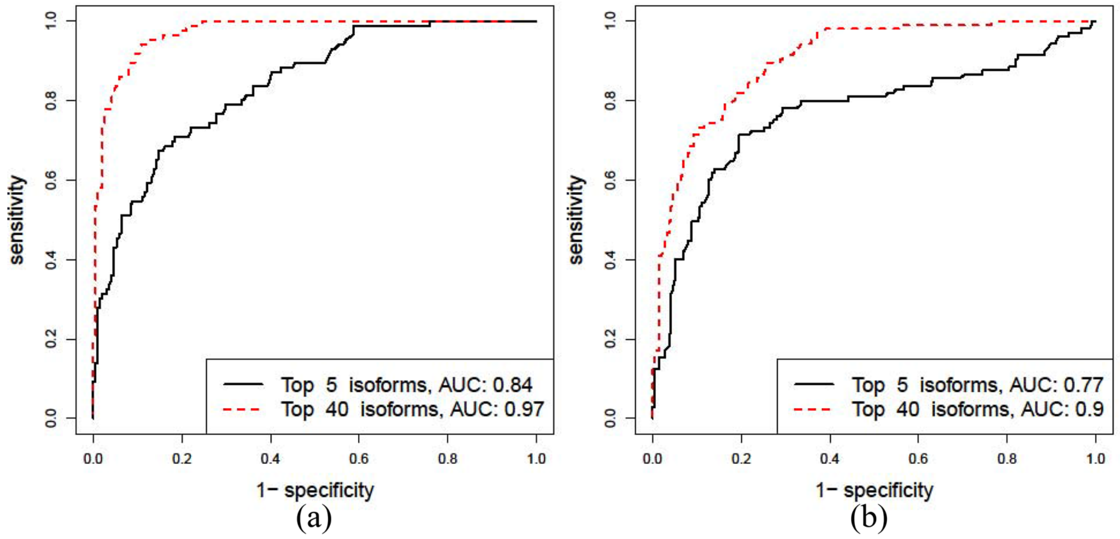

Supplementary Figure S6: ROC curve for top 5 and 40 isoforms from lncRNA isoforms in a. discovery set and **b.** validation set.

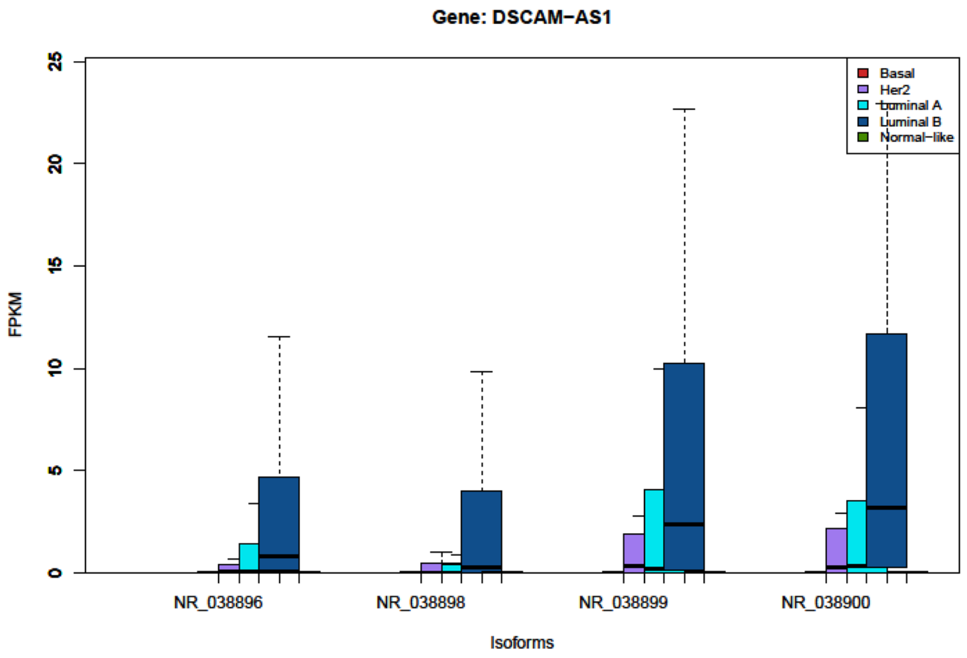

Supplementary Figure S7: Boxplots of isoforms of gene DSCAM-AS1.

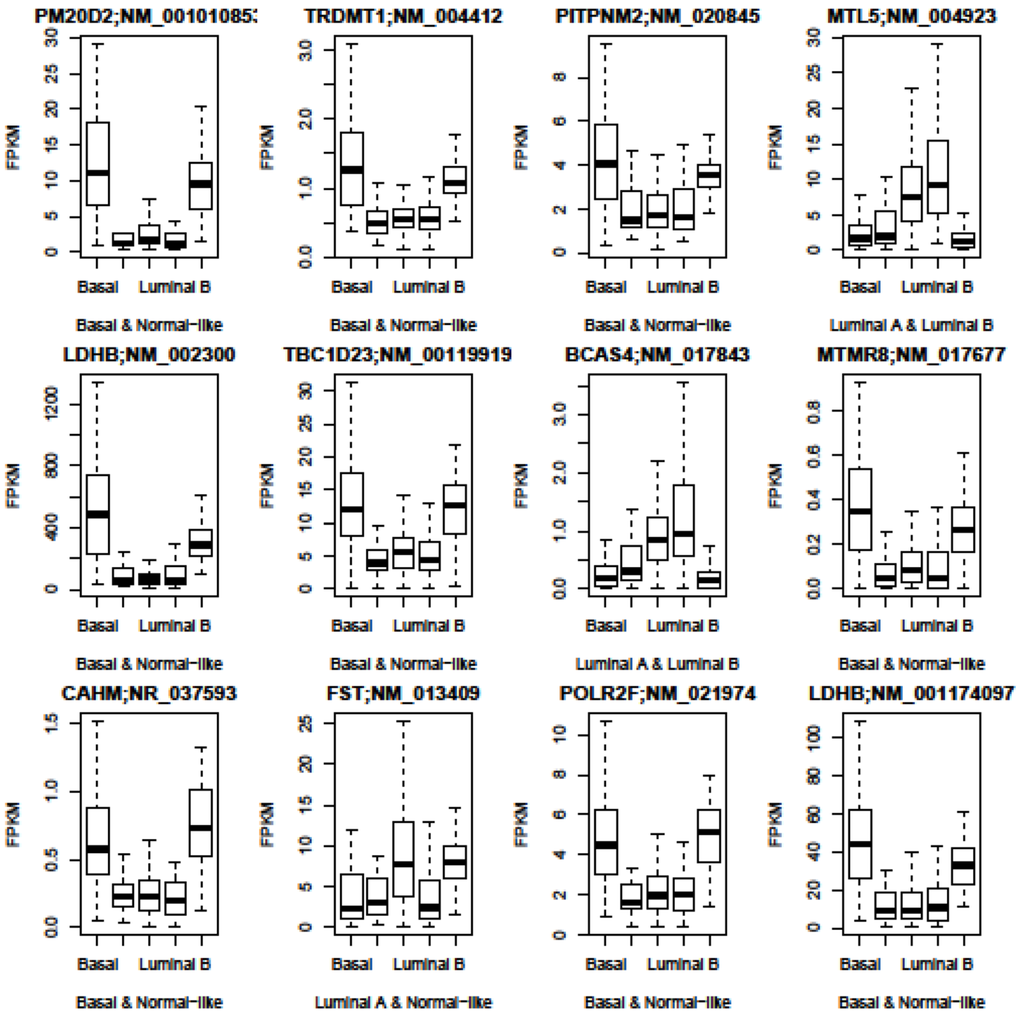

Supplementary Figure S8: Boxplots of the top 12 subtype-coexpression isoforms. On the x-axis, the subtypes follow the alphabetical order of Basal-like, Her2, Luminal A, Luminal B and Normal-like.

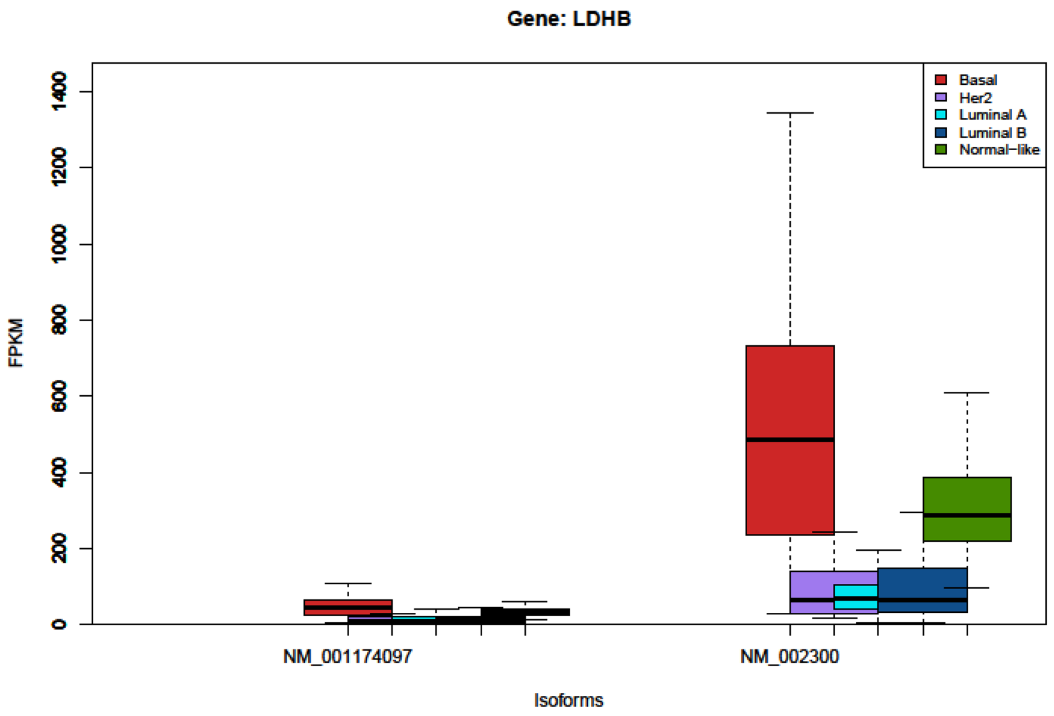

Supplementary Figure S9: Boxplots of isoforms of gene LDHB.

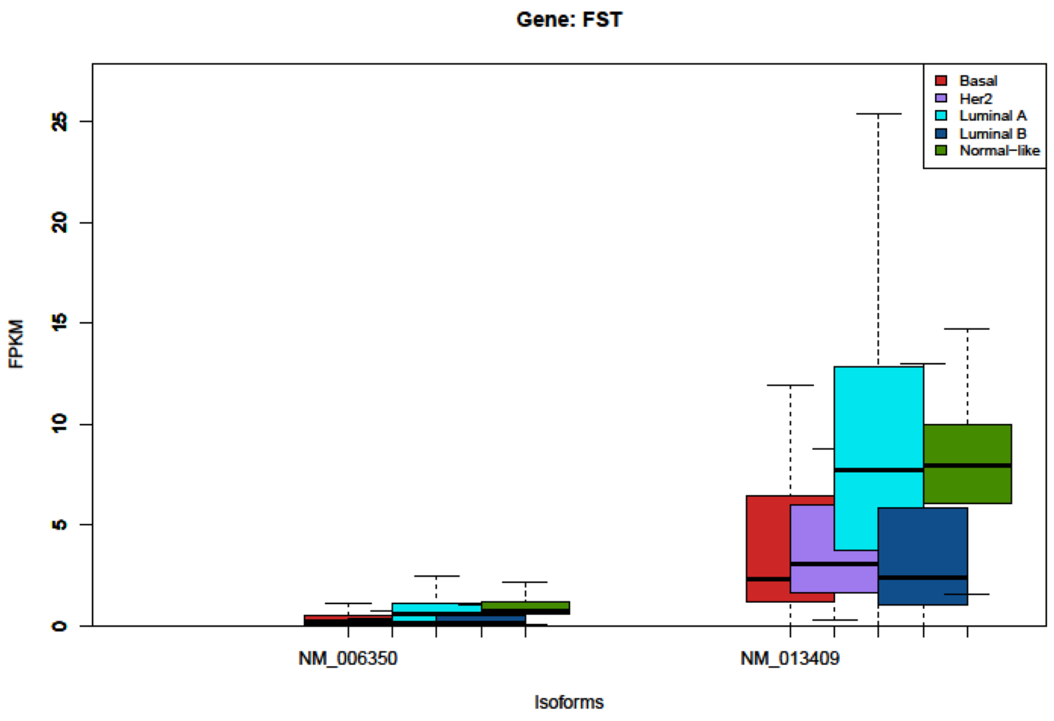

Supplementary Figure S10: Boxplots of isoforms of gene FST.

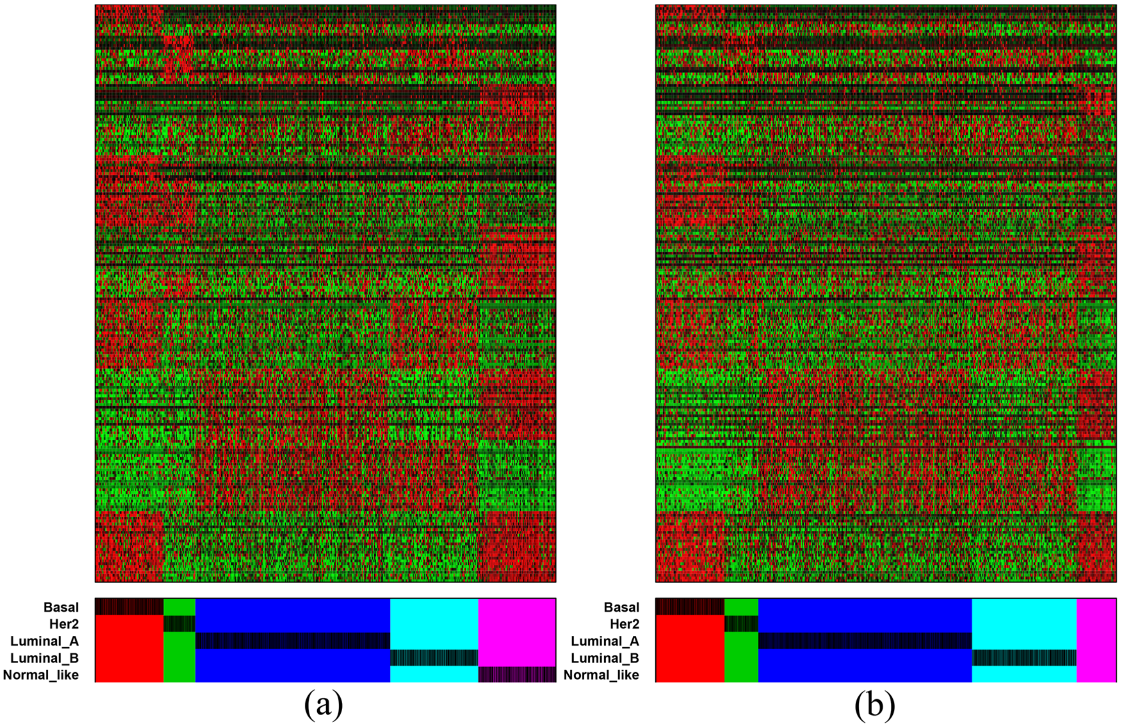

Supplementary Figure S11: Color map of top 203 two-subtype-specific isoforms from (a) the discovery and (b) validation sets. Red and green indicate expression levels above and below median, respectively. The isoforms in each subtype are ordered by AUC from bottom to top and right to left.

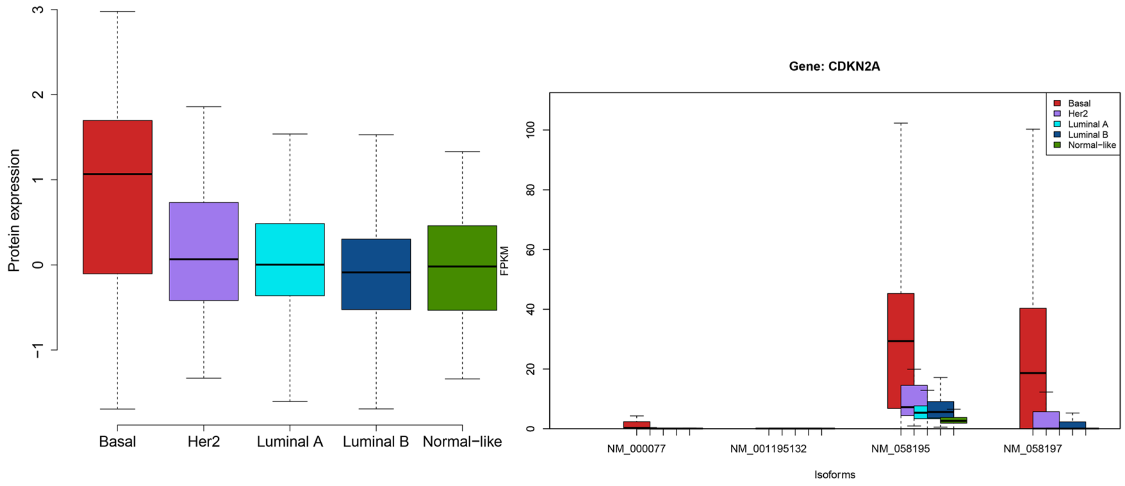

(a) (b)

Supplementary Figure S12: Expression at protein level a. and isoform level **b.** of CDKN2A gene. Isoform NM_058197 and the protein are specific to Basal subtype.

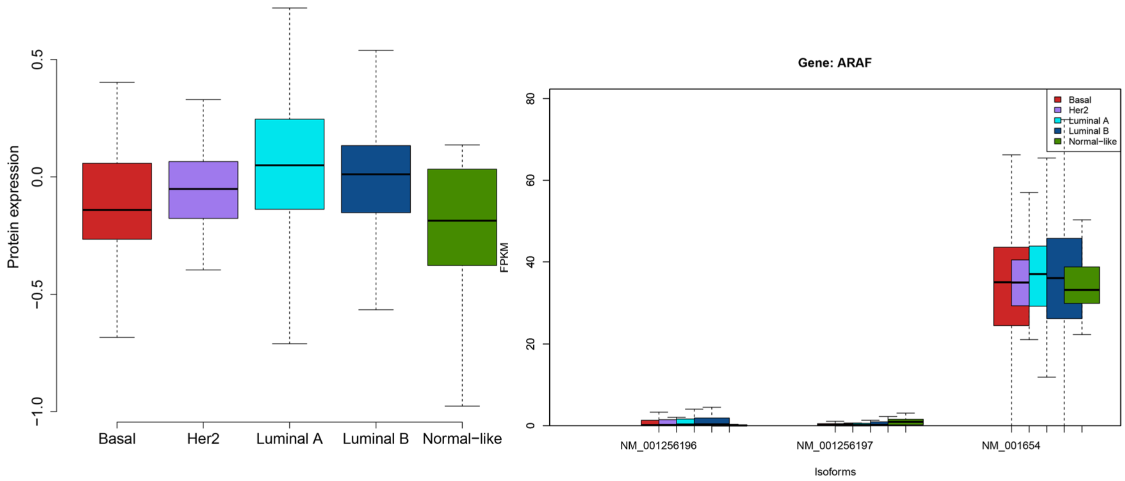

(a) (b)

Supplementary Figure S13: Expression at protein level a. and isoform level **b.** of ARAF gene. Isoform NM_001256196 and the protein are specific to Luminal A subtype.

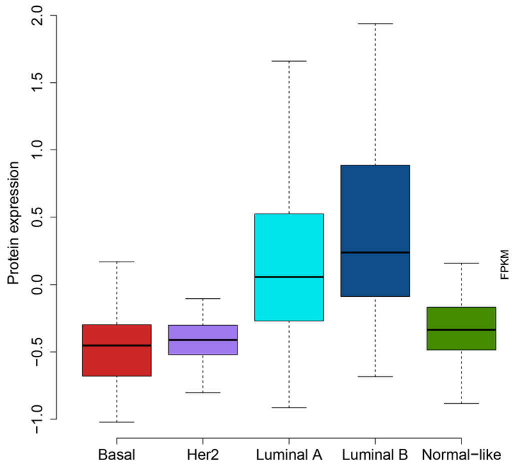

(a)

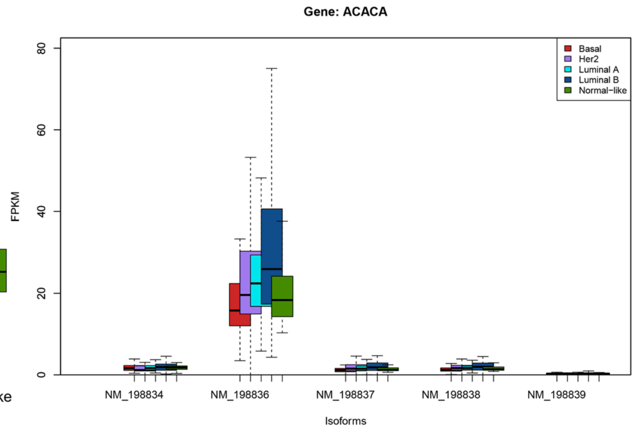

(b)

Supplementary Figure S14: Expression at protein level a. and isoform level **b.** of ACACA gene. Isoform NM_198837, NM_198838 and the protein are specific to Luminal B subtype.

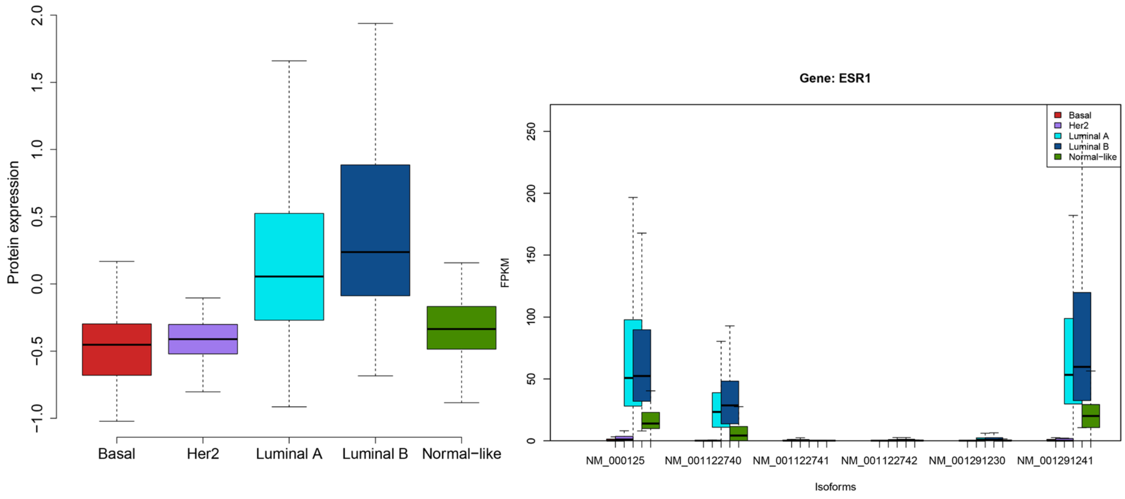

(a) (b)

Supplementary Figure S15: Expression at protein level a. and isoform level **b.** of ESR1 gene. Isoform NM_001122742 and the protein are co-expressed to Luminal A subtype and Luminal B subtype. For visual purpose, isoform expression is scaled to log2(FPKM+1).

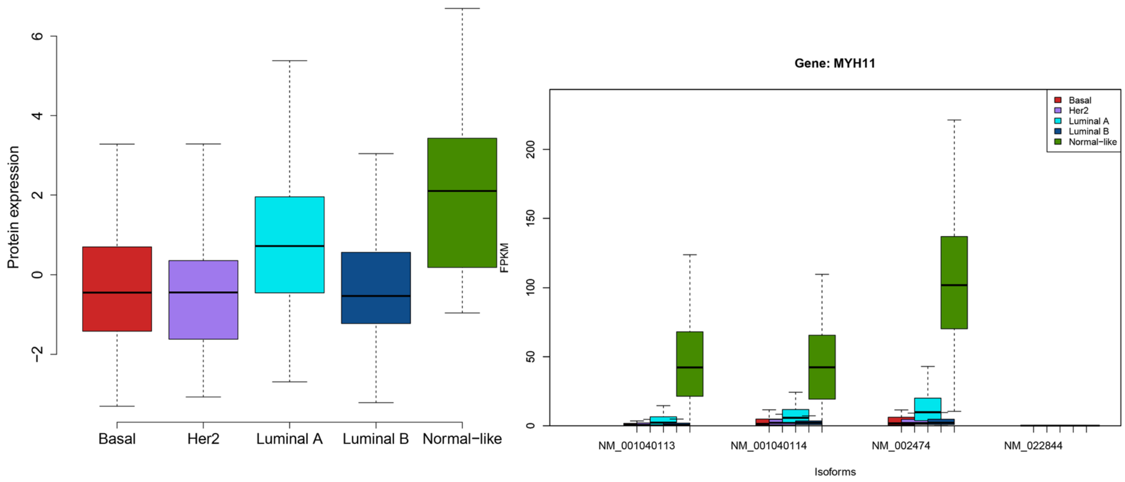

(a) (b)

Supplementary Figure S16: Expression at protein level a. and isoform level **b.** of MYH11 gene. Three isoforms NM_001040113, NM_001040114, NM_002474 and the protein are co-expressed to Luminal A subtype and Normal subtype. For visual purpose, isoform expression is scaled to log2(FPKM+1).

Supplementary Table S1: Distribution of the tissue samples across the study centers

Discovery set		Validation set	
**Tissue source**	**Sample**	**Tissue source**	**Sample**
UCSF	190	Walter Reed	87
Christiana Healthcare	70	Indivumed	80
Cureline	62	International Genomics Consortium	77
UNC	42	MSKCC	64
Mayo	37	Duke	48
University of Pittsburgh	29	Roswell Park	41
ILSBio	14	Asterand	32
Greater Poland Cancer Center	6	University of Miami	16
Ontario Institute for Cancer Research (OICR)	1	ABS - IUPUI	14
		MD Anderson	4
Total	451	Total	463

Supplementary Table S2: Distribution of subtypes in discovery set, validation set and protein validation set

Subtype	Discovery set	Validation set	Protein validation set
Basal	67	69	115
Her2	31	34	60
Luminal A	191	215	313
Luminal B	86	105	163
Normal-like	76	40	17

Supplementary Table S3: Median AUCs of isoform-level and gene-level gene expression for classifying the molecular subtypes in the discovery set and the validation set

Dataset	Isoform level	Gene level
Discovery set	0.65	0.67
Validation set	0.62	0.64

Supplementary Table S4: Number of isoforms selected from top 10% AUC differences between isoform- and gene-level in the discovery set and validated in the validation sets

Subtype	Discovered	Validated
Basal	103	68
Her2	15	5
Luminal A	91	39
Luminal B	90	47
Normal-like	247	121

Supplementary Table S5: Number of subtype-coexpression isoforms

Subtypes	# Isoforms
Basal & Her2	29
Basal & Luminal A	11
Basal & Luminal B	54
Basal & Normal-like	369
Her2 & Luminal A	6
Her2 & Luminal B	11
Her2 & Normal-like	35
Luminal A & Luminal B	358
Luminal A & Normal-like	586
Luminal B & Normal-like	41
